# Influence of Different Warming Methods in Rabbits Subjected to Prolonged Pneumoperitoneum

**DOI:** 10.3390/ani15192891

**Published:** 2025-10-03

**Authors:** Rodrigo N. A. Curopos, José A. Damasceno-Ferreira, Francisco J. B. Sampaio, Diogo Benchimol de Souza

**Affiliations:** 1 Urogenital Research Unit, Rio de Janeiro State University, Rio de Janeiro 20551-030, Brazil; 2 Department of Veterinary Pathology and Clinic, Federal Fluminense University, Niterói 24230-321, Brazil

**Keywords:** hypothermia, laparoscopy, minimally invasive surgery, perioperative care, pneumoperitoneum, warming methods

## Abstract

**Simple Summary:**

Pneumoperitoneum is associated with hypothermia in prolonged laparoscopic procedures, especially in small dogs and cats. In this study, the use of a forced-air warming system (combined or not with the use of heated CO_2_) reduced the heat loss in a rabbit model. This information may reduce surgical morbidity and improve the perioperative recovery in small animal hospitals.

**Abstract:**

Objective: The objective of this study was to evaluate the influence of heated CO_2_ and forced-air warming on perioperative temperature in a rabbit model of prolonged pneumoperitoneum. Methods: Thirty-seven New Zealand rabbits, weighing an average of 3.85 kg, were divided into four experimental groups with different warming methods: the control group (CT) underwent unheated pneumoperitoneum (22 °C); another group (HP) underwent pneumoperitoneum with heated CO_2_ (36 °C); a third group (FA) underwent unheated pneumoperitoneum but with the use of a forced-air warming device (43 °C) positioned between the animal and the surgical table; and the fourth group of animals (HP + FA) underwent heated pneumoperitoneum with the use of a forced-air warming device. For all animals, the pneumoperitoneum was maintained for 120 min. The animals’ temperature was measured immediately before sedation (I0), at the beginning of insufflation (I1, which was mandatory 60 min after I0), every 15 min during pneumoperitoneum (I2–I9), and 15 min after desufflation (I10). The data were analyzed and compared by Student’s *t*-test, ANOVA, Pearson’s correlation and linear regression, considering *p* < 0.05 as significant. Results: There was no difference between the groups regarding weight, temperature at I0, temperature at I1, volume of CO_2_ used, or Δt (I0–I1). In all groups, there was a decrease in temperature when comparing the final instants (I9 or I10) with the initial instants (I0 or I1) of the study (*p* < 0.05). However, the groups that used the forced-air warming (FA and HP + FA) had a smaller decrease in temperature and a higher final temperature, with no difference between these groups. Furthermore, these groups recovered their temperature better after deflation (from I9 to I10). For all groups, a correlation between time and temperature was observed, but in the groups that used a heated mattress, the slope of the linear regression line was smaller. Conclusions: The use of a forced-air warming system (combined or not with the use of heated CO_2_) reduced the heat loss during prolonged pneumoperitoneum in a small animal model. This warming method is recommended for preventing hypothermia in laparoscopic surgeries with expected prolonged surgical time.

## 1. Introduction

Perioperative hypothermia represents a critical and frequently underestimated complication in veterinary surgery, with its prevalence varying across procedural types. In dogs, moderate to severe hypothermia is reported in more than 30% of cases in which conventional open surgical procedures are performed [1], which already warrants considerable clinical attention. Preliminary data suggest that this issue is exacerbated in laparoscopic surgery, where the incidence of moderate to severe hypothermia may exceed 50% [2]. This high incidence is particularly concerning given the overall goal of minimally invasive surgeries to provide improved patient care and optimal surgical recovery.

There are inherent factors of laparoscopic procedures that may contribute to this enhanced heat loss. One of these factors is the insufflation of carbon dioxide gas into the abdominal cavity, necessary for creating an intra-abdominal working space. Pneumoperitoneum is routinely created with cold CO_2_, which promotes heat loss through convection and evaporation from the visceral surface area [3]. Furthermore, the prolonged surgical duration often associated with complex laparoscopic procedures also exacerbates the total heat deficit [4,5].

Indeed, veterinary surgery has seen significant advancements with the integration of laparoscopic techniques. Historically limited to routine procedures such as ovariectomy and cryptorchidectomy, the scope of minimally invasive surgery in dogs and cats has expanded considerably. In the last decade, the feasibility and benefits of advanced laparoscopy for applications such as adrenalectomy [6,7], cholecystectomy [8], and partial nephrectomy [9], which were once considered the exclusive domain of open surgery, have been demonstrated. For these procedures, strong evidence associates laparoscopy with reduced postoperative pain, shorter hospitalization times, and faster recovery rates compared to open approaches. However, with more complex laparoscopic procedures being performed, longer surgical times are expected. These combined factors highlight a persistent challenge in preventing hypothermia during laparoscopic surgeries.

Perioperative hypothermia, even in mild forms, is associated with a range of detrimental physiological consequences that can significantly impair patient recovery and increase morbidity. Hypothermia directly inhibits enzyme activity, leading to impaired coagulation, and increased blood loss [10]. Furthermore, it can prolong the effects of anesthetic agents by reducing their metabolism and excretion, thereby delaying postanesthetic recovery [11]. Compromised thermoregulation also suppresses immune function, increasing susceptibility to surgical site infections and other nosocomial complications [12]. Cardiovascular effects, such as vasoconstriction and arrhythmias, are important alterations observed especially in moderate and severe hypothermia and contribute to adverse outcomes [13]. These multifaceted impacts underscore the critical importance of maintaining normothermia throughout the perioperative period to optimize patient safety and improve surgical outcomes.

As many patients who undergo laparoscopic procedures in veterinary practice are small breed dogs or cats, the risk of perioperative hypothermia is even greater. Animals with a high surface area-to-volume ratio experience disproportionately greater heat loss through radiation, convection, and evaporation compared to larger animals, even under similar environmental conditions [14,15]. Furthermore, the limited thermal insulation provided by thinner subcutaneous fat layers and smaller muscle mass further exacerbates this vulnerability. Consequently, these animals are more susceptible to rapid decreases in core body temperature, necessitating meticulous thermoregulatory management strategies to mitigate this elevated risk [16].

The use of warmed and humidified carbon dioxide for insufflation has emerged as a key strategy in thermoregulatory management during laparoscopic surgery in children [17]. In the veterinary literature, the results are controversial, with an experimental study showing no benefits of using warmed and humidified carbon dioxide during laparoscopy [18]. More recently, a clinical study demonstrated that employing heated CO_2_ effectively reduces intraoperative heat loss, leading to higher core body temperatures in dogs submitted to laparoscopic ovariectomy compared to using unheated gas [19]. This intervention minimizes the temperature gradient between the insufflated gas and the peritoneal cavity, thereby diminishing heat transfer away from the patient [20].

Beyond interventions directly targeting the insufflated gas, external warming techniques such as forced-air warming systems play a complementary and crucial role in preventing perioperative hypothermia during laparoscopic surgery. These devices circulate warm air over the patient’s surface, effectively counteracting heat loss through convection and radiation [21]. Forced-air warming blankets are highly effective in transferring heat to the patient, contributing to the maintenance of core body temperature throughout the procedure, and are routinely used for open and laparoscopic surgeries [22]. Combined with internal warming strategies like heated CO_2_ insufflation, forced heated air provides a comprehensive approach that may optimize patient thermoregulation and minimize perioperative hypothermia.

The hypothesis of the present study is that the use of heated pneumoperitoneum, forced-air warming system, or the combination of both methods may reduce the heat loss associated with pneumoperitoneum in a small animal model. As few studies have investigated methods for preventing perioperative hypothermia associated with pneumoperitoneum, especially for longer procedures, this study supplies missing information that can guide clinical practice. Thus, the objective of the present study was to evaluate the influence of heated CO_2_ and forced-air warming, independently or combined, on perioperative temperature in a rabbit model of prolonged pneumoperitoneum.

## 2. Materials and Methods

Thirty-seven New Zealand rabbits weighting 3.85 ± 0.69 Kg were used in this study. Animals were obtained from a commercial rabbit farm kept in the Urogenital Research Unit animal facilities with a controlled temperature (20 °C ± 1 °C) and artificial dark–light cycles (lights on from 7:00 a.m. to 7:00 p.m.) and had free access to standard rabbit chow and water in individual cages. This project was formally approved by the local ethics committee under the protocol number CEUA-018/2024 and followed national and international regulations on animal experimental use.

The animals were randomly assigned into four experimental groups as follows: control group (CT, n = 9); heated pneumoperitoneum group (HP, n = 10); forced-air warming group (FA, n = 10); heated pneumoperitoneum and forced-air warming group (HP + FA, n = 9). The number of animals per group followed pilot studies of our group.

At the experiment day, animals received pre-anesthetic medication (acepromazine 0.05 mg/Kg, ketamine 5 mg/Kg and midazolam 0.5 mg/Kg intramuscularly), and an intravenous catheter was placed for Ringer’s lactate solution (at room temperature) administration (3 mL/Kg/h). Anesthesia was induced with propofol (10 mg/kg, intravenously) and maintained with isoflurane in oxygen administered through endotracheal intubation. The ventral abdomen, 10cm around the umbilical scar, was prepared for aseptic surgery.

Hasson technique was used to introduce the first portal on the umbilical scar. This was used to establish the pneumoperitoneum of six millimeters of mercury with CO_2_ flow rate set to two liters per minute. The insufflator used (CM-40L, Confiance Medical, Rio de Janeiro, Brazil) allowed us to turn on or off the gas heating, and this was set accordingly to the experimental group. Groups HP and HP + FA received heated (36.5 °C) CO_2_ insufflation, while groups CT and FA received cold (22 °C) CO_2_.

All animals were positioned above a forced-air warming blanket which was connected to the forced-air warming device (DL3000, Delta Life, São Paulo, Brazil). For the groups FA and HP + FA, the device was set to blow 43 °C warmed air, while for groups CT and HP, the device was turned off throughout the experiments.

**Figure 1:**
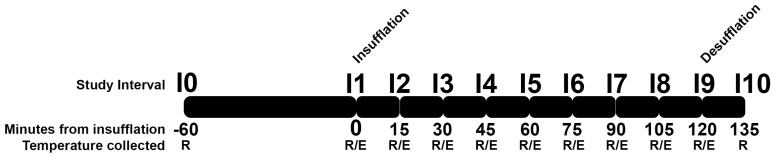
Graphical representation of the different instants of data collection. R: Rectal; E: Esophageal. The study started at I0 with rectal temperature being measured. At I1 (60 min after I0), the abdomen insufflation started and was maintained until I9, with rectal and esophageal temperatures being measured at each 15 min interval. After 15 min from desufflation (I10), rectal temperature was measured and the experiment ends.

The animals remained under general anesthesia and pneumoperitoneum for 120 min. During this period, the body temperature was measured by a digital rectal probe (1301430, G-Tech, São Paulo, Brazil) and an esophageal probe (placed at the cervical esophagus) coupled to a multiparametric monitor (DL 1000, Delta Life, São José dos Campos, Brazil). Rectal temperatures (RTs) and esophageal temperatures (ETs) were measured in Celsius degrees at each 15 min interval from the beginning of insufflation (reported as I1) until the desufflation after 120 min (reported as I9). RT was also measured just before the pre-anesthetic medication administration (reported as I0), which was standardized to 60 min before the insufflation. Further, RT was also measured 15 min after desufflation (reported as I10). The timepoints of data collection are illustrated in Figure 1.

Immediately after I9 (120 min after insufflation), the cavity was desuflatted, isoflurane administration was interrupted, and the cavity and skin were sutured routinely. Animals were extubated, received analgesics and allowed to recover from anesthesia.

All data was considered normally distributed by Kolmogorov–Smirnov test and the residuals were tested by a Q-Q Plot, also being considered normally distributed. The initial (rectal and esophageal) temperatures, body weight and CO_2_ volume were compared among the groups tested by using one-way ANOVA. Also, one-way ANOVA with Tukey’s post-test was used for comparing the final (at I9, for esophageal; at I9 and I10, for rectal) temperatures. Student’s *t*-test was used to compare the initial versus final temperature for each group, and for comparing final temperature between CT and HP groups. Finally, Pearson’s correlation and linear regression analyses were performed between the temperatures and the experimental timepoints. Results were considered significant when *p* < 0.05. All analyses were performed with the GraphPad Prism 10.3.0 software (GraphPad Software, San Diego, CA, USA). No animals were excluded from the study and all data were used for the analyses. Power analysis was used to confirm the adequacy of the sample size used in the study and confirmed power above 0.9 for the parameters analyzed. All results were presented as mean ± standard deviation.

## 3. Results

The groups were considered homogeneous at the beginning of the experiment as body weight, initial RT (I0 and I1), and initial ET (I1) were considered similar (*p* > 0.05) among the four groups. Also, the temperature reduction during the animal’s preparation (from I0 to I1) was similar among the groups. Additionally, the CO_2_ volume used for abdominal insufflation was similar (*p* > 0.05) among the groups. These data are presented in Table 1.

For all experimental groups, we observed a significant temperature reduction during the study. When comparing the RT obtained at the final experimental intervals (I9 or I10) with those from the initial intervals (I0 or I1), all groups presented statistically significant differences (*p* < 0.05). These data are presented in Table 2.

**Figure 2:**
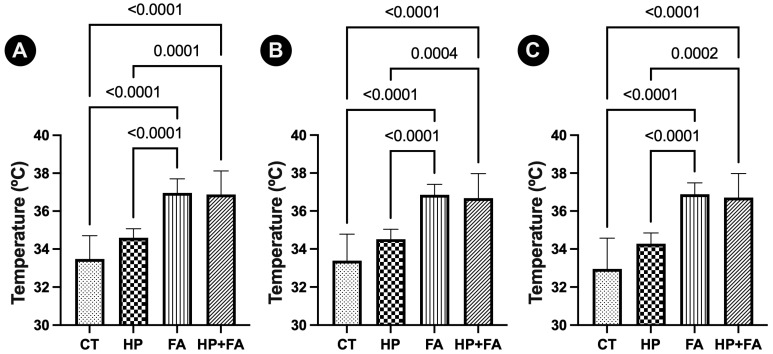
Graphical representation of temperatures of rabbits after 120 min of pneumoperitoneum with different warming methods. Results show that animals that used the forced-air warming device (groups FA and HP + FA) had higher final temperatures than groups that did not use the forced-air warming device. Graphic (**A**) represents the esophageal temperatures measured at I9. Graphic (**B**) depicts the rectal temperatures measured at I9. Graphic (**C**) depicts the rectal temperatures collected at I10. Both data shows significant differences (*p* < 0.05) between groups FA or HP + FA and groups CT or HP.

When comparing the final temperatures (I9 or I10) among the experimental groups, it was observed that groups that used the forced-air warming device (groups FA and HP + FA) had higher values than groups that did not used the forced-air warming device. These results are represented in Figure 2. The temperature reduction during the study (Δt (I1–I9) and Δt (I1–I10) was also smaller in groups that used the forced-air warming device (groups FA and HP + FA) than in groups CT and HP. These results are presented in Table 3.

We also compared the temperature difference in the immediate recovery period (immediately after abdominal desufflation). For this, the Δt (I9-I10) was compared among the groups. This analysis showed that groups CT and HP continued with temperature reductions while groups FA and HP + FA (even with the forced-air warming device being turned off at I9) showed temperature increasing. This result is presented in Table 3.

**Figure 3:**
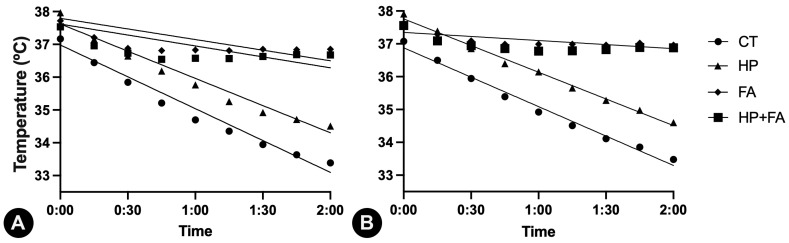
Graphical representation of the correlations and linear regressions of time and temperatures of rabbits submitted to 120 min of pneumoperitoneum with different warming methods. Results show that animals that used the forced-air warming device (groups FA and HP + FA) had less inclined linear equations, denoting slower temperature reductions in relation to time, in comparison to groups CT or HP. (**A**) Rectal temperatures × time; (**B**) esophageal temperatures × time.

For all experimental groups, there were significant negative correlations between time and temperatures (both rectal and esophageal), although R2 for groups FA and HP + FA was <0.65. This indicates that, independent of the group, as time passes by, the temperature reduces. This was confirmed as regression models were significant for all groups when analyzing the rectal temperature × time. Groups FA and HP + FA showed less inclined curves. When analyzing the linear regression of esophageal temperature x time, group HP + FA showed *p* > 0.05, while other groups confirmed the results obtained for rectal temperature analysis. These data are presented in Table 4 and Figure 3.

Further, when comparing the final temperatures (I9) of groups CT and HP, it was observed that group HP showed a higher rectal and esophageal temperature (1.32 °C, *p* = 0.0378 and 1.12 °C, *p* = 0.0205, respectively) than control animals.

## 4. Discussion

The present study shows that pneumoperitoneum is associated with temperature reductions and reinforces the necessity of using hypothermia preventive methods for animals of small body mass, especially when prolonged surgical times are expected. The results indicate approaches that can provide superior safety and better recovery for surgical patients, contributing to the improvement of veterinary surgical practices.

The rabbit has been used as an experimental model for comparison to small animals with success [23,24]. The animals used in the present study had comparable body weights (3.85 Kg in mean) to cats and small dogs, which have been increasingly submitted to laparoscopic procedures. The use of the rabbit model brings some limitations as the dense coat of rabbits may prevent heat changes with external environment, in contrast to dogs and cats. On the other hand, the use of dogs or cats for this experimental study would not be allowed by our local ethics committee while purpose-breed rabbits are seen as laboratory animals [25]. 

The homogeneous distribution of animals across the experimental groups, with similar body weight, initial temperatures and volume of CO_2_ used, supports the studied variables being the sole differing parameters, and strengthens the internal validity of the study findings. This meticulous control ensures that the observed outcomes can be directly attributed to the variations in the heating methods investigated [26]. Consequently, these results provide a clear and reliable insight into the specific impact of each warming technique, minimizing the influence of confounding factors and allowing for a precise evaluation of their efficacy.

Despite the different warming methods employed, the study findings consistently demonstrate a significant reduction in animals’ temperature after 120 min of pneumoperitoneum when compared to initial values. This reduction, regardless of the heating strategy, suggests that prolonged pneumoperitoneum exerts an important physiological impact which should not be neglected. This observation highlights the persistent challenge of preventing hypothermia during prolonged minimally invasive procedures and emphasizes the need for investigation into strategies to counteract the temperature decline [19].

The present study observed that the forced-air warming device significantly impacted the thermal profiles of the animals. Specifically, groups utilizing the forced-air warming mattress achieved a higher final body temperature and smaller temperature reduction, compared to groups where no such warming device was employed. This sustained thermal support likely contributed to the improved outcomes observed in these groups, highlighting the efficacy of forced-air warming in preventing perioperative hypothermia, which is in accordance with previous studies [22,27,28].

The most drastic reductions in the mean body temperatures of cats and dogs during anesthesia occurs within the initial 20 min following induction [29], which is considered the first phase for development of hypothermia. In this phase, a rapid decrease in central body temperature occurs as a redistribution of heat from the warm central compartment to cooler peripheral tissues [27,30]. For preventing the temperature reduction of this initial phase, thermal blankets are little effective [31]. Indeed, in the current study the use of forced-air warming device did not prevent the initial temperature drop (until I4) but was effective in avoiding temperature reductions after 45 min of pneumoperitoneum (I4) until the end of the study (I9 or I10). Forced-air warming systems reduce heat loss by increasing the skin’s microenvironmental temperature to a more physiological level. This effectively eliminates the thermal gradient between the patient and the environment, thereby slowing the rate of heat flow away from the body [32,33]. Delivering warm air to a patient’s skin significantly reduces heat loss through radiation and convection [32]. This is a crucial mechanism for the second phase of anesthesia-associated heat loss, as these types of heat loss play a major role in the development of intraoperative hypothermia [27].

Groups CT and HP (without the use of forced-air warming device) did not achieve the third phase of anesthetic-induced hypothermia, when a thermal steady state was observed (where heat production equals heat loss), throughout the study [27]. Even after the desufflation and anesthetic administration, the temperature of these groups continued to drop (from I9 to I10). This is presumably explained by the continuous heat loss that was occurring during all instants of the experiment. Meanwhile, groups FA and HP + FA achieved almost stable temperatures (within 36.5–36.9 °C) from I4 until the end of the study. Even with the equipment turned off at I9, the body temperature continued to rise gradually.

A well-known correlation between anesthetic time and temperature decline [34] was confirmed in all studied groups of this study. This finding reinforces the importance of avoiding unnecessary prolongation of anesthetic and surgical time. Conversely, our results also indicate that moderate (or even severe) hypothermia can be reached within conventionally accepted surgical durations in some groups. Based on the linear regression of rectal temperature x time, the time to reach moderate (36.4 °C) and severe (33.9 °C) hypothermia [1,35] was estimated (considering a 95% confidence interval). According to the model obtained, group CT should reach moderate hypothermia between 7.7 and 26.7 min, and severe hypothermia between 86.4 and 105.7 min after the insufflation. The group receiving heated pneumoperitoneum should reach moderate hypothermia between 40.4 and 47.9 min, and severe hypothermia between 128.5 and 141.4 min after the insufflation. Group FA should reach moderate hypothermia between 111.2 and 155.6 min, and severe hypothermia between 302.5 and 453.9 min after the insufflation. Finally, group HP + FA should reach moderate hypothermia between 86.5 and 149.9 min, and severe hypothermia between 257.7 and 499.5 min after the insufflation.

According to these estimated times for moderate and severe hypothermia, dogs undergoing laparoscopic ovariectomy, a procedure that can be performed in 20 to 150 min [36,37], would experience moderate or severe hypothermia after surgery if no warming method is used. If only heated pneumoperitoneum is used, animals within the median surgical time would experience moderate hypothermia after surgery, and those with prolonged surgical times would experience severe hypothermia. On the other hand, for animals using forced-air warming, moderate hypothermia would be present only in those with prolonged surgical times, and severe hypothermia could be avoided even at the longest surgical time.

For more prolonged procedures, such as laparoscopic adrenalectomy with mean surgical times from 138 to 158 min [4,5], moderate hypothermia would be expected even if forced-air warming is used. Among the tested methods, forced-air warming would be advised as the only method to prevent severe hypothermia in such longer procedures.

From the study findings, no beneficial effects in body temperature were observed when using both (heated pneumoperitoneum and forced-air warming) methods. The groups FA and HP + FA showed similar results, and different results from CT and HP groups, showing that the forced-air device made a significant difference among the groups. It is reasonable to suppose that combining these two warming techniques could be beneficial, as they prevent different heat loss pathways, and in distinct anatomical areas, potentially offering a more complete approach to maintaining body temperature. The only manuscript that compared these methods showed a synergistic effect of using both [38]. Indeed, the mentioned study used pigs as experimental models and had a different experimental design and equipment, warranting future studies to further research the potential benefits of using both warming methods.

Finally, one may suppose that the use of heated pneumoperitoneum would be ineffective for preventing perioperative hypothermia during laparoscopic procedures. This would align with a previous study conducted in experimental, purpose-breed dogs [18], and could be explained as heating CO_2_ does not prevent heat loss from the external environment, only attenuating the heat loss due to using the standard cold and CO_2_ [38]. However, when directly comparing groups CT and HP, it was observed that heated peritoneum exerted some preventive effects, with more than 1 °C of difference among these groups in the final temperature, which is in accordance with a recent study conducted in a clinical scenario [19]. From these findings, it is possible to state that heated pneumoperitoneum exerts some preventive effects on temperature reduction, which is limited when compared to forced-air warming systems.

This study had some limitations. Since no intracavitary surgical maneuvers were performed during the experiment, no introduction and removal of instruments were necessary, and gas leakage from the portals was significantly minimized. Additionally, blood loss was virtually eliminated, as there was no intraabdominal incision. This approach fundamentally differs from the clinical scenario and could substantially impact temperature reduction outcomes, reducing the direct reproducibility on clinical settings. Furthermore, physiological and anatomical differences between the species used as experimental model and canine and feline patients may limit the direct translatability of these results to the clinical setting. Even so, the present study brings important information directing to better understanding on hypothermia prevention for laparoscopic procedures in small animals. Future studies are warranted to better elucidate the role of warming methods for larger patients, and on clinical scenarios.

## 5. Conclusions

The use of a forced-air warming system (combined or not with the use of heated CO_2_) reduced the heat loss during prolonged pneumoperitoneum in a small animal model. This warming method is recommended for preventing hypothermia in laparoscopic surgeries in small patients when the surgical time is expected to be greater than 45 min. The use of heated CO_2_ alone had limited effects for preventing perioperative hypothermia in small patients, being useful in combination with forced-air warming system or for procedures with surgical times less than 40 min.

Combined warming methods could still provide substantial benefits during longer and more complex surgeries, and this potential should be explored further in future research.

## Figures and Tables

**Table 1:** Comparison of initial data among the rabbits submitted to pneumoperitoneum with different warming methods.

	CT	HP	FA	HP + FA	*p* Value
Body weight (Kg)	3.74 ± 0.76	3.88 ± 0.68	3.90 ± 0.79	3.77 ± 0.64	0.9524
Rectal temperature—I0 (°C)	39.26 ± 0.30	39.44 ± 0.36	39.42 ± 0.36	39.36 ± 0.43	0.7270
Rectal temperature—I1 (°C)	37.17 ± 1.34	37.97 ± 0.27	37.72 ± 0.64	37.53 ± 0.78	0.2609
Δt-r (I0–I1) (°C)	2.09 ± 1.19	1.48 ± 0.42	1.70 ± 0.54	1.82 ± 0.64	0.3923
Esophageal temperature—I1 (°C)	37.08 ± 1.22	37.91 ± 0.36	37.62 ± 0.61	37.56 ± 0.78	0.1991
CO_2_ volume (L)	12.9 ± 6.43	10.7 ± 18.6	28.3 ± 39.9	3.61 ± 1.02	0.1606

CT: Control group; HP: heated pneumoperitoneum group; FA: forced-air warming group; HP + FA: heated pneumoperitoneum and forced-air warming group; Δt-r (I0–I1): difference in rectal temperature from I0 and I1. Means compared by one-way ANOVA. Data presented as mean ± standard deviation.

**Table 2:** Comparison of initial (I0 or I1) and final (I9 or I10) rectal temperatures of rabbits submitted to pneumoperitoneum with different warming methods.

	Initial Temperature (I0 or I1)	Final Temperature (I9 or I10)	*p* Value
Group CT			
I0 vs. I10 (°C)	39.26 ± 0.30	32.96 ± 1.61	<0.0001
I0 vs. I9 (°C)	39.26 ± 0.30	33.39 ± 1.39	<0.0001
I1 vs. I10 (°C)	37.17 ± 1.34	32.96 ± 1.61	<0.0001
I1 vs. I9 (°C)	37.17 ± 1.34	33.39 ± 1.39	<0.0001
Group HP			
I0 vs. I10 (°C)	39.44 ± 0.36	34.28 ± 0.57	<0.0001
I0 vs. I9 (°C)	39.44 ± 0.36	34.51 ± 0.53	<0.0001
I1 vs. I10 (°C)	37.97 ± 0.27	34.28 ± 0.57	<0.0001
I1 vs. I9 (°C)	37.97 ± 0.27	34.51 ± 0.53	<0.0001
Group FA			
I0 vs. I10 (°C)	39.42 ± 0.36	36.89 ± 0.62	<0.0001
I0 vs. I9 (°C)	39.42 ± 0.36	36.85 ± 0.56	<0.0001
I1 vs. I10 (°C)	37.72 ± 0.64	36.89 ± 0.62	0.0126
I1 vs. I9 (°C)	37.72 ± 0.64	36.85 ± 0.56	0.0070
Group HP + FA			
I0 vs. I10 (°C)	39.36 ± 0.43	36.71 ± 1.27	<0.0001
I0 vs. I9 (°C)	39.36 ± 0.43	36.68 ± 1.29	<0.0001
I1 vs. I10 (°C)	37.53 ± 0.78	36.71 ± 1.27	0.0025
I1 vs. I9 (°C)	37.53 ± 0.78	36.68 ± 1.29	0.0022

CT: Control group; HP: heated pneumoperitoneum group; FA: forced-air warming group; HP + FA: heated pneumoperitoneum and forced-air warming group; means compared by Student’s *t*-test. Data presented as mean ± standard deviation.

**Table 3:** Comparison of final temperatures (I9 or I10) and temperature reductions during the study among the rabbits submitted to pneumoperitoneum with different warming methods.

	CT	HP	FA	HP + FA	*p* Value
Rectal temperature—I9 (°C)	33.39 ± 1.39	34.51 ± 0.53	36.85 ± 0.56 ^a,b^	36.68 ± 1.29 ^a,b^	<0.0001
Rectal temperature—I10 (°C)	32.96 ± 1.61	34.28 ± 0.57	36.89 ± 0.62 ^a,b^	36.71 ± 1.27 ^a,b^	<0.0001
Esophageal temperature—I9 (°C)	33.48 ± 1.22	34.60 ± 0.47	36.96 ± 0.78 ^a,b^	36.88 ± 1.25 ^a,b^	<0.0001
Δt-r (I1–I9) (°C)	3.78 ± 0.89	3.45 ± 0.48	0.87 ± 0.90 ^a,b^	0.85 ± 0.65 ^a,b^	<0.0001
Δt-r (I1–I10) (°C)	4.21 ± 1.06	3.69 ± 0.50	0.83 ± 0.98 ^a,b^	0.82 ± 0.64 ^a,b^	<0.0001
Δt-e (I1–I9) (°C)	3.60 ± 0.95	3.31 ± 0.26	0.66 ± 0.94 ^a,b^	0.67 ± 0.65 ^a,b^	<0.0001
Δt-r (I9–I10) (°C)	0.43 ± 0.33	0.23 ± 0.18	−0.04 ± 0.10 ^a,b^	−0.03 ± 0.10 ^a,b^	<0.0001

CT: Control group; HP: heated pneumoperitoneum group; FA: forced-air warming group; HP + FA: heated pneumoperitoneum and forced-air warming group; Δt-r (I1–I9): difference in rectal temperature from I1 and I9. Δt-r (I1–I10): difference in rectal temperature from I1 and I10. Δt-e (I1–I9): Difference in esophageal temperature from I1 and I9. Means compared by one-way ANOVA with Tukey’s post-test. ^a^: different from group CT; ^b^: different from group HP. Data presented as mean ± standard deviation.

**Table 4:** Correlations and Linear regression data of time and temperatures (rectal and esophageal) of rabbits submitted to pneumoperitoneum with different warming methods.

	CT		HP		FA		HP + FA	
Pearsons’s Correlation	R^2^	*p* Value	R^2^	*p* Value	R^2^	*p* Value	R^2^	*p* Value
Time (min) × Rectal temperature (°C)	0.9818	<0.0001	0.9782	<0.0001	0.6249	0.0019	0.5966	0.0027
Time (min) × Esophageal temperature (°C)	0.9866	<0.0001	0.9928	<0.0001	0.5905	0.0078	0.4598	0.0223
Linear regression								
Time (min) × Rectal temperature (°C)	0.6729	<0.0001	0.9013	<0.0001	0.4046	<0.0001	0.2301	<0.0001
Equation	RT = −0.03237 × t + 36.98		RT = −0.02766 × t + 37.62		RT = −0.01079 × t + 37.80		RT = −0.0111 × X + 37.62	
Time (min) × Esophageal temperature (°C)	0.4938	<0.0001	0.8220	<0.0001	0.0584	0.0217	0.0231	0.1757
Equation	ET = −0.02986 × t + 36.88		ET = −0.02709 × t + 37.76		ET = −0.00412 × t + 37.35		*	

CT: Control group; HP: heated pneumoperitoneum group; FA: forced-air warming group; HP + FA: heated pneumoperitoneum and forced-air warming group. RT: Rectal temperature; ET: esophageal temperature; t: time. * Equation not presented as *p* > 0.05.

## Data Availability

The raw data supporting the conclusions of this article will be made available by the authors on request.
